# Flexible and Accurate Calibration Method for Non-Overlapping Vision Sensors Based on Distance and Reprojection Constraints

**DOI:** 10.3390/s19214623

**Published:** 2019-10-24

**Authors:** Tao Jiang, Xu Chen, Qiang Chen, Zhe Jiang

**Affiliations:** 1 School of Automation and Electrical Engineering, University of Science and Technology Beijing, Beijing 100083, China; 2 Beijing Institute of Aerospace Control Devices, Beijing 100854, China; xuchen8917@163.com (X.C.); qiangchen1980@163.com (Q.C.); jiangzhe9596@163.com (Z.J.)

**Keywords:** calibration, nonoverlapping vision sensors, bundle adjustment

## Abstract

This paper addresses the problem of flexible and accurate global calibration for multiple non-overlapping vision sensors in a confined workspace. Instead of using an auxiliary calibration pattern, the proposed method uses one laser tracker and only its accessory target sphere to obtain all the 3D calibration points and then accomplish the initial estimation of pose between the vision sensors. Then, the 3D calibration points and the extrinsic parameters between vision sensors are further optimized via the bundle adjustment algorithm based on the distance and reprojection constraints. Experiments were conducted to validate the performance and the experimental results demonstrate that the distance error can be decreased from 3.5 mm to 0.8 mm after introducing the distance and reprojection constraints.

## 1. Introduction

Accurate calibration of the relative pose between vision sensors is crucial for the final measurement accuracy of multi-vision-sensor (MVS) measurement systems. Although there are many existing methods that can efficiently handle the precise pose calibration problem for vision sensors with a common field-of-view (FOV), more effort is needed in the study of flexible and accurate calibration methods for non-overlapping vision sensors. Since the intrinsic calibration of a single vision sensor has some comprehensive solutions such as the methods presented by Tsai [1] and Zhang [2], we now focus on the extrinsic parameter calibration for vision sensors without overlapping FOVs in this paper.

According to the difference of auxiliary tools used in the calibration process, the published extrinsic calibration methods for non-overlapping vision sensors can be roughly classified into three categories. The first approach utilizes high precision measuring instruments to establish a global reference coordinate and finishes the global calibration by coordinate transformation. Lu and Li [3] employed two theodolites and additional calibration targets to accomplish the global calibration. To solve the problem of the blind observation zone of a dual theodolite, Zhang et al. [4] utilized one theodolite and one planar calibration target to obtain the 3D coordinates and corresponding image coordinates of the feature points. Xie et al. [5] used a high-resolution digital camera instead of a theodolite as a global measurement device to complete global calibration. Similarly, Dong et al. [6] realized the extrinsic calibration of a camera network based on close-range photogrammetry. In addition, Liu et al. [7] used a laser tracker to establish a global coordinate system, and then used one precise three dimensional target to obtain the rotation and translation matrix of each local vision sensor coordinate system relative to the global laser tracker coordinate system, which in turn, realized the global calibration among multiple sensors. Additionally, Lu et al. [8] utilized a coordinate measuring machine to accomplish the calibration of stereo cameras.

The second approach does not need auxiliary measuring instruments, but uses customized calibration targets that contain precise spatial geometric information. Taking advantage of a one-dimensional target, Liu et al. [9] proposed a global calibration method for multiple sensors based on the principle of cross-ratio invariance and collinear constraints of the calibration object. Likewise, vanishing points of a one-dimensional target were also used for solving the coordinates of each target point in camera coordinate systems [10]. Liu et al. [11] further used the laser rangefinder instead of a long one-dimensional target for the calibration of vision sensors in a wide range. In order to improve the acquisition efficiency of feature points for calibration, Zhang et al. [12] formed a dual-planar target by fixing two planar calibration panels on the two ends of a rigid beam. During the calibration process, each camera saw the planar target in its own FOV, and the invariance of spatial structure between the two planar targets was fully utilized to calculate the relative pose between two cameras. Additionally, Gong et al. [13] constructed a global target covered with circular features to realize the quick calibration of multiple vision sensors for some specific applications. It is also worth noting that Kumar et al. [14] skillfully acquired the calibration image of the same target by means of planar mirroring. The global calibration of multiple sensors was implemented by making the transformation between the virtual local vision sensor coordinate system and the real one. Liu et al. [15] designed a three-dimensional target, which was combined of three planar targets to calibrate the relative orientation between multiple depth cameras. Ni et al. [16] used the Lie algebra optimization to address the relative pose estimation for multiple cameras in the context of motion-based camera calibration. In their methods, a planar target is almost indispensable.

The final approach does not use any auxiliary tools during the calibration process, which is also called self-calibration methods. This kind of method is used to accomplish the calibration of the relative pose between vision sensors based on the invariance of the spatial structure in the observed scenario. Specifically, Equivel et al. [17] applied the structure from motion algorithm to locate the change of the relative position of each image sequence acquired by each vision sensor and then implemented the global calibration of multiple sensors based on rigid constraints among the sensors. Lebraly et al. [18] also presented a similar algorithm. Anjum et al. [19] improved the estimation robustness of the relative orientation and position by taking the unobserved trajectory and the exit–entrance direction of each object into consideration. In addition, Mendikute et al. [20] presented a self-calibration technique for vision systems by using redundant information of machine measurements to avoid extra mechanical anchoring or calibration means, but the method lacks versatility.

Among the aforementioned methods, the third kind of approach is the most flexible, but also has the worst calibration repeatability and accuracy. Essentially, most of self-calibration methods are not designed for measurement applications. Regarding calibration methods using customized targets, they can achieve moderate calibration accuracy and have a wide range of applications. However, the second kind of method is usually executed under controllable test environments such as in laboratories or factories. Despite the high cost of auxiliary measuring instruments, the first approach is still popular in an outdoor environment due to its high precision and strong capacity of resisting disturbance.

In some practical measurement applications, the field of view of each vision sensor is so narrow that there is no space to place such a large planar target or similar 3D target. The presented method in this paper was designed to overcome the calibration difficulties on these occasions. Unlike existing approaches, our method does not need additional targets to locate feature points for calibration. The accessorial target sphere of the laser tracker is used to form those calibration points. Since the target sphere diameter can range from 10 mm to about 50 mm, the accurate 3D locations of calibration points can be easily obtained in most constrained environments by freely moving the small target sphere at several positions. By substituting a small optical target sphere for those planar or three-dimensional targets, our method can accomplish the extrinsic calibration of vision sensors installed in a confined workspace. Despite the fact that the accurate locations of the target sphere center in the global reference frame can be directly read out by the laser tracker, the recovered 3D positions in the local camera coordinate are inevitably affected by noises. Thus, the distance and reprojection constraints were introduced in our method to restrain the noise influence. The remainder of this paper is organized as follows. Section 2 introduces the basic calibration principle and procedure using a laser tracker and its accessorial target sphere. In Section 3, the bundle adjustment for extrinsic parameters based on distance and projection constraints is described in detail. Section 4 presents a physical experiment conducted to verify the feasibility of the proposed approach, specifically, the accuracy improvement due to bundle adjustment based on distance and projection constraints. Conclusions are drawn in Section 5.

## 2. Calibration Principle

**Figure 1:**
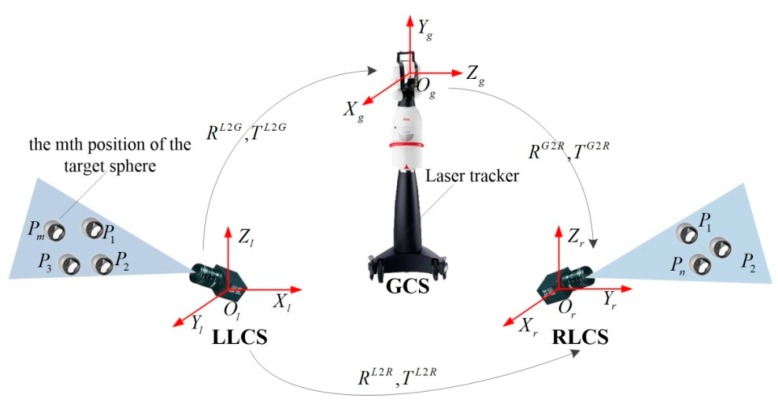
Illustration of the calibration procedure for two non-overlapping cameras.

Since the local coordinate system of a vision sensor is always consistent with its camera coordinate, we hereinafter refer to the camera coordinate frame as the coordinate frame of the vision sensor. Without loss of generality, the global calibration of multiple vision sensors without overlapping FOVs is represented by the calibration of two non-overlapping cameras in this section. As illustrated in Figure 1, the relative pose between the left and right cameras is to be calibrated. For brevity, the global coordinate system established by the laser tracker is denoted by global coordinate system (GCS), and local coordinate systems (LCS) of the left and right cameras are denoted by LLCS and RLCS, respectively.

The basic calibration procedure contains three steps. First, the target sphere is placed at several (at least three) different positions and then observed by the camera. The three dimensional coordinate of the sphere center in the GCS can be easily read out from the laser tracker and its coordinate in the LCS can be reconstructed according to the known radius of the sphere and its pixel coordinate in the projection image. Second, the relative position and orientation between the LCS and the GCS can be calculated after the point correspondence is given in the first step. Finally, the relative pose between the LLCS and the RLCS is computed by rigid transformation.

### 2.1. 3D Localization of the Target Sphere Center in the LCS and the GCS

At each placement position, the coordinate of the target sphere center in the GCS can be read directly from the laser tracker. Meanwhile, the coordinate of the target sphere center in the LCS can be reconstructed through the projection contour of the target sphere on the image plane, according to Shiu et al. [21].

Specifically, the equation of the ellipse projected on the image plane by the target sphere is as follows:

(1) $$au^{2}+bv^{2}+cuv+du+ev+f=0$$

where (u, v) is the pixel coordinate of the point on the image ellipse. The coefficient (a–f) of the ellipse can be calculated by elliptic fitting after extracting the contour of the image ellipse. Suppose (x, y, z) is the back-projection coordinate of the point on the image ellipse in the LCS and f0 is the focal length of the camera, then Equation (1) can be rewritten as:

(2) $$Ax^{2}+By^{2}+Cxy+Dxz+Eyz+Fz^{2}=0$$

where $A\;=\;af_{0}^{2}$, $B\;=\;af_{0}^{2}$, $C\;=\;cf_{0}^{2}$, $D\;=\;df_{0}$, $E\;=\;ef_{0}$, F = f. Equation (2) can be further expressed in terms of a quadratic form:

(3) $$\left[\begin{array}{ccc} x & y & z \end{array}\right]Q{\left[\begin{array}{ccc} x & y & z \end{array}\right]}^{T}=0;Q=\left[\begin{array}{ccc} A & C/2 & D/2\\ C/2 & B & E/2\\ D/2 & E/2 & F \end{array}\right]$$

At the ith position, the three-dimensional coordinate P c i of the target sphere center in the LCS is shown below:

(4) $$P_{i}^{c}=\left[\begin{array}{c} P_{ix}^{c}\\ P_{iy}^{c}\\ P_{iz}^{c} \end{array}\right]=R_{0}\sqrt{\frac{\left|\lambda_{1}\right|\left(\left|\lambda_{2}\right|+\left|\lambda_{3}\right|\right)}{\left|\lambda_{3}\right|\left(\left|\lambda_{1}\right|+\left|\lambda_{3}\right|\right)}}\left[\begin{array}{c} e_{3x}\\ e_{3y}\\ e_{3z} \end{array}\right]$$

where {λ1, λ2, λ3} is the eigenvalue of matrix Q in Equation (3), and e3 = (e3x, e3y, e3z)T is the eigenvector of matrix Q corresponding to the eigenvalue λ3. Furthermore, R0 is the radius of the target sphere. It can be seen from Equations (3) and (4) that the three-dimensional coordinates of the target sphere centers in the LCS are closely related to the size of the target sphere and its position relative to the camera.

### 2.2. Local Calibration of the Relative Pose between the LCS and the GCS

Once the three-dimensional coordinates of the target sphere center in the LCS and the GCS at several placements are known, the local calibration of the relative pose between the LCS and the GCS can be accomplished through the following two steps. In the first step, three positions of the target sphere center are randomly selected to establish a transfer coordinate system (TCS) and obtain the initial value of the transformation matrix from the LCS to the GCS. Then, all positions of the target sphere center are involved in the optimization of the transformation matrix using a nonlinear iterative method such as the well-known Levenberg–Marquardt algorithm.

Assume that the coordinates of the target sphere center at three different positions in the LCS (LLCS or RLCS) are $P_{1}^{c}$, $P_{2}^{c}$, $P_{3}^{c}$ respectively, and their counterparts in the GCS are $P_{1}^{g}$, $P_{2}^{g}$, and $P_{3}^{g}$. As long as the three points are not on a straight line, we can establish a TCS as follows:

(1) The origin O_t_ of the TCS in the LCS and the GCS are shown below:
  (5) $$\{\begin{array}{c} O_{t}^{c}\left(\begin{array}{ccc} O_{tx}^{c} & O_{ty}^{c} & O_{tz}^{c} \end{array}\right)=\frac{1}{3}\sum_{i=1}^{3}{\left(\begin{array}{ccc} x_{i}^{c} & y_{i}^{c} & z_{i}^{c} \end{array}\right)}^{T}\\ O_{t}^{g}\left(\begin{array}{ccc} O_{tx}^{g} & O_{ty}^{g} & O_{tz}^{g} \end{array}\right)=\frac{1}{3}\sum_{i=1}^{3}{\left(\begin{array}{ccc} x_{i}^{g} & y_{i}^{g} & z_{i}^{g} \end{array}\right)}^{T} \end{array}$$
  where ($x_{i}^{c}$, $y_{i}^{c}$, $z_{i}^{c}$)T = $P_{i}^{c}$($x_{i}^{c}$, $y_{i}^{c}$, $z_{i}^{c}$) and ($x_{i}^{g}$, $y_{i}^{g}$, $z_{i}^{g}$)^T^ = $P_{i}^{g}$($x_{i}^{g}$, $y_{i}^{g}$, $z_{i}^{g}$) for i = 1, 2, and 3.
(2) The x-axis Xt of the TCS in the LCS and the GCS are represented by:
  (6) $$\{\begin{array}{c} X_{t}^{c}\left(\begin{array}{ccc} X_{tx}^{c} & X_{ty}^{c} & X_{tz}^{c} \end{array}\right)=\frac{{\left(P_{1}^{c}-O_{t}^{c}\right)}^{T}}{{\left\|P_{1}^{c}-O_{t}^{c}\right\|}_{2}}\\ X_{t}^{g}\left(\begin{array}{ccc} X_{tx}^{g} & X_{ty}^{g} & X_{tz}^{g} \end{array}\right)=\frac{{\left(P_{1}^{g}-O_{t}^{g}\right)}^{T}}{{\left\|P_{1}^{g}-O_{t}^{g}\right\|}_{2}} \end{array}$$
(3) The z-axis Zt of the TCS in the LCS and the GCS are:
  (7) $$\{\begin{array}{c} Z_{t}^{c}\left(\begin{array}{ccc} Z_{tx}^{c} & Z_{ty}^{c} & Z_{tz}^{c} \end{array}\right)=\frac{{\left(P_{2}^{c}-P_{1}^{c}\right)}^{T}\times {\left(P_{3}^{c}-P_{1}^{c}\right)}^{T}}{{\left\|{\left(P_{2}^{c}-P_{1}^{c}\right)}^{T}\times {\left(P_{3}^{c}-P_{1}^{c}\right)}^{T}\right\|}_{2}}\\ Z_{t}^{g}\left(\begin{array}{ccc} Z_{tx}^{g} & Z_{ty}^{g} & Z_{tz}^{g} \end{array}\right)=\frac{{\left(P_{2}^{g}-P_{1}^{g}\right)}^{T}\times {\left(P_{3}^{g}-P_{1}^{g}\right)}^{T}}{{\left\|{\left(P_{2}^{g}-P_{1}^{g}\right)}^{T}\times {\left(P_{3}^{g}-P_{1}^{g}\right)}^{T}\right\|}_{2}} \end{array}$$
(4) The y-axis Yt of the TCS can be calculated by means of the cross product of Xt and Zt, that is:
  (8) $$\{\begin{array}{c} Y_{t}^{c}\left(\begin{array}{ccc} Y_{tx}^{c} & Y_{ty}^{c} & Y_{tz}^{c} \end{array}\right)=X_{t}^{c}\times Z_{t}^{c}\\ Y_{t}^{g}\left(\begin{array}{ccc} Y_{tx}^{g} & Y_{ty}^{g} & Y_{tz}^{g} \end{array}\right)=X_{t}^{g}\times Z_{t}^{g} \end{array}$$

According to Equation (5), the translation vectors $T_{t}^{c}$ and $T_{t}^{g}$ from the TCS to the LCS and the GCS are respectively given by:

(9) $$\{\begin{array}{c} T_{t}^{c}\left(\begin{array}{ccc} T_{tx}^{c} & T_{ty}^{c} & T_{tz}^{c} \end{array}\right)=O_{t}^{c}\left(\begin{array}{ccc} O_{tx}^{c} & O_{ty}^{c} & O_{tz}^{c} \end{array}\right)\\ T_{t}^{g}\left(\begin{array}{ccc} T_{tx}^{g} & T_{ty}^{g} & T_{tz}^{g} \end{array}\right)=O_{t}^{g}\left(\begin{array}{ccc} O_{tx}^{g} & O_{ty}^{g} & O_{tz}^{g} \end{array}\right) \end{array}$$

Based on Equations (6)–(8), the rotation matrices $R_{t}^{c}$ and $R_{t}^{g}$ from the TCS to the LCS and the GCS can be calculated as follows:

(10) $$R_{t}^{c}=\left[\begin{array}{ccc} X_{tx}^{c} & Y_{tx}^{c} & Z_{tx}^{c}\\ X_{ty}^{c} & Y_{tx}^{c} & Z_{tx}^{c}\\ X_{tz}^{c} & Y_{tx}^{c} & Z_{tx}^{c} \end{array}\right];R_{t}^{g}=\left[\begin{array}{ccc} X_{tx}^{g} & Y_{tx}^{g} & Z_{tx}^{g}\\ X_{ty}^{g} & Y_{tx}^{g} & Z_{tx}^{g}\\ X_{tz}^{g} & Y_{tx}^{g} & Z_{tx}^{g} \end{array}\right]$$

Now, we can calculate the relative pose (that is, the rotation matrix $R_{c}^{g}$ and the translation vector $T_{c}^{g}$) between the LCS and the GCS using Equations (9) and (10):

(11) $$\left\{ \begin{array}{l} R_{c}^{g}=R_{t}^{g}R_{c}^{t}=R_{t}^{g}{\left(R_{t}^{c}\right)}^{T}\\ T_{c}^{g}=R_{t}^{g}T_{c}^{t}+T_{t}^{g}=-R_{t}^{g}{\left(R_{t}^{c}\right)}^{T}T_{t}^{c}+T_{t}^{g}=-R_{c}^{g}T_{t}^{c}+T_{t}^{g} \end{array}\right.$$

Using the rotation matrix and the translation vector given by Equation (11) as the initial value, the relative pose can be further optimized by minimizing the objective function defined by

(12) $$\underset{{\left(R_{c}^{g},T_{c}^{g}\right)}^{*}}{\arg\min}{\sum_{i}\left\|P_{i}^{g}-\hat{m}\left(R_{c}^{g},T_{c}^{g},P_{i}^{c}\right)\right\|}^{2}$$

where

(13) $$\hat{m}\left(R_{c}^{g},T_{c}^{g},P_{i}^{c}\right)={\hat{P}}_{i}^{g}={\hat{R}}_{c}^{g}P_{i}^{c}+{\hat{T}}_{c}^{g}$$

### 2.3. Global Calibration of the Relative Pose between Vision Sensors

Suppose the rotation matrix and the translation vector between the LLCS and the GCS are $R_{l}^{g}$ and $T_{l}^{g}$, and their counterparts between the RLCS and the GCS are $R_{r}^{g}$ and $T_{r}^{g}$, the rotation matrix $R_{l}^{r}$ and the translation vector $T_{l}^{r}$ can be given by:

(14) $$\left\{ \begin{array}{l} R_{l}^{r}=R_{g}^{r}R_{l}^{g}={\left(R_{r}^{g}\right)}^{T}R_{l}^{g}\\ T_{l}^{r}=R_{g}^{r}T_{l}^{g}+T_{g}^{r}={\left(R_{r}^{g}\right)}^{T}\left(T_{l}^{g}-T_{r}^{g}\right) \end{array}\right.$$

where *$R_{l}^{g}$, $T_{l}^{g}$,* $R_{r}^{g}$, and $T_{r}^{g}$ can be obtained using the method described in Section 2.2. So far, the global calibration of extrinsic parameters between two vision sensors has been accomplished after two local calibrations have been carried out.

## 3. Bundle Adjustment Based on Distance and Reprojection Constraints

In Section 2, we described the global calibration procedure for multiple vision sensors without an overlapping field of view in detail. Nevertheless, the practical image of the target sphere usually deteriorates to some extent due to the variations of the environmental illumination and the observation angle of the vision sensor. Furthermore, the uncertainties of the feature extraction during the sphere image processing and the elliptic fitting process also introduce errors into the final three-dimensional location of the target sphere center. Position errors of the target sphere center in the LCS will inevitably reduce the extrinsic calibration accuracy.

In order to decrease the adverse impact imported by the deviation of the three-dimensional positions of the target sphere in the LCS, two constraints are taken into consideration. One is the distance between different positions of the target sphere center in the GCS, and the other is involved with the image projection of the target sphere center besides that of the sphere contour.

Assume that the three-dimensional coordinates of the target sphere center in the GCS and the LCS at the ith position are $P_{i}^{g}$ and $P_{i}^{c}$, respectively, and the deviation vector between the reconstructed coordinates of the target sphere center and the real ones at the ith position in the LCS is Δ$P_{i}^{c}$. Then, we can optimize the local calibration by bundle adjustment based on the distance and projection constraints and the objective function is given by:

(15) $$\min\left\{\sum_{i=1}^{N}{\left\|P_{i}^{g}-\hat{m}\left(R_{c}^{g},T_{c}^{g},P_{i}^{c}-\Delta P_{i}^{c}\right)\right\|}^{2}+\sum_{i=1}^{i=N-1}\sum_{j=i+1}^{j=N}{\left\|D_{ij}^{c}-D_{ij}^{g}\right\|}^{2}+\sum_{i=1}^{N}d_{i}^{2}+\sum_{i=1}^{N}e_{i}^{2}\right\}$$

where $D_{ij}^{c}$ = ||$P_{i}^{c}$ − Δ$P_{i}^{c}$ − $P_{j}^{c}$ + Δ$P_{j}^{c}$||, $D_{ij}^{g}$ = ||$P_{i}^{g}$ − $P_{j}^{g}$|| and ||$D_{ij}^{c}$ − $D_{ij}^{g}$||^2^ represents the distance constraint between the three-dimensional coordinates of target sphere centers in the LCS and the GCS. The item $d_{i}^{2}$ represents the distance between the image projection point of the target sphere center and the major axis of the projection ellipse of the target sphere, and $e_{i}^{2}$ represents the reprojection errors of the reconstructed target sphere center positions. Assume that the camera intrinsic parameters *f_x_*, *f_y_*, u_0_, and *v*_0_, the distortion parameters *k*_1_ and *k*_2_ are known, and the linear equation of the major axis of the projection ellipse of the target sphere is *l*(*l*_1_,−1,*l*_2_), then *d_i_* can be given by:

(16) $$d_{i}=\left\|l^{T}\hat{m}\left(f_{x},f_{y},u_{0},v_{0},k_{1},k_{2},P_{i}^{c}-\Delta P_{i}^{c}\right)\right\|$$

Based on the conclusion of Sun et al. [22], the image projection point of the target sphere center should be located on the major axis of the projection ellipse of the target sphere, that is, the ideal value of *d_i_* should be zero. Additionally, the principal point of the image should be on the major axis of the projection ellipse of the target sphere, according to Daucher et al. [23]. Then, we can obtain the linear equation of the major axis using the known principal point of the image (*u*_0_, *v*_0_) and the geometric center of the projection ellipse (*u_c_*, *v_c_*) as follows:

(17) $$\left\{ \begin{array}{c} l_{1}=\left(v_{0}-v_{c}\right)/\left(u_{0}-u_{c}\right)\\ l_{2}=v_{0}-l_{1}u_{0}=v_{c}-l_{1}u_{c} \end{array}\right.$$

where the coordinates of the geometric center of the projection ellipse are given by:

(18) $$\left\{ \begin{array}{c} u_{c}=\left(2bd-ce\right)/\left(c^{2}-4ab\right)\\ v_{c}=\left(2ae-cd\right)/\left(c^{2}-4ab\right) \end{array}\right.$$

and the coefficients a, b, c, d, and e are defined in Equation (1).

Additionally, the error function ei represents the distance between the extracted ellipse feature point (*u_ij_,v_ij_*) and the re-projection ellipse at the ith position, which is defined as follows:

(19) $$e_{i}=\sum_{j=1}^{M}\left\|\hat{m}\left(u_{ij},v_{ij},f_{0},R_{0},P_{i}^{c}-\Delta P_{i}^{c}\right)\right\|$$

where M represents the number of extracted feature points on the ith projection image. Given the known target sphere center ($P_{i}^{c}$ − Δ$P_{i}^{c}$), the radius of the sphere *R*_0_ and the focal length *f*_0_, the re-projection error *e_i_* can be easily calculated by the method proposed by Shiu et al. [21].

During the bundle adjustment process, *Δ$P_{i}^{c}$* is set to zero as its initial value, and other parameters can be initialized using the method described in Section 2. From Equations (15)–(19), the optimal estimation of the rotation matrix and translation vector between the LCS and the GCS can be obtained by means of the large-scale trust-region reflective algorithm. After the precise local calibration, the precise calibration between vision sensors can be realized using the method described in Section 2.3.

## 4. Experimental Results

**Figure 2:**
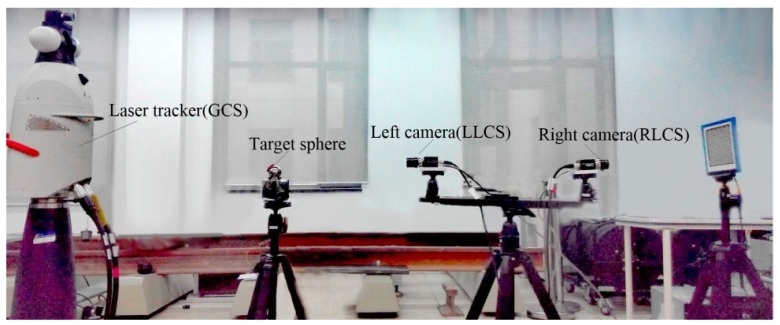
Experimental layout for the global calibration of two non-overlapping cameras.

To verify the feasibility of the proposed method, a typical vision measurement system with two non-overlapping vision sensors was established, as illustrated in Figure 2. The two vision sensors were both AVT GC1380H digital cameras with 12 mm Schneider lens. The image resolutions of the vision sensors were 1360 × 1024. The reference GCS was built on a Leica API-T3 laser tracker and the radius of its target sphere was 19.5 mm. The LLCS and RLCS coincided with the corresponding local camera coordinates, respectively.

During the experiment, the target sphere was moved several times (no less than three) within the FOV of each camera. The target sphere should be placed as far as possible to cover the measurement range of each camera. For each position of the target sphere, the laser tracker was used to take 10 samples on the coordinates of the sphere center with the center-of-mass of the sphere center coordinates as the precise coordinates of the target sphere center in the GCS, thus reducing the location positioning noise of the target sphere center. Similarly, for each position of the target sphere, the camera also captured 10 projection images of the target sphere to reduce the influence of illumination on the image quality.

In the following section, the ellipse detection and fitting results in the projection images of the target sphere are first introduced. Then, the 3D positions of the target sphere center in the LCS and the GCS at each position are given. Finally, the accuracy of the global calibration results before and after the bundle adjustment are analyzed and compared.

### 4.1. Ellipse Detection and Fitting in the Projection Image of the Target Sphere

**Figure 3:**
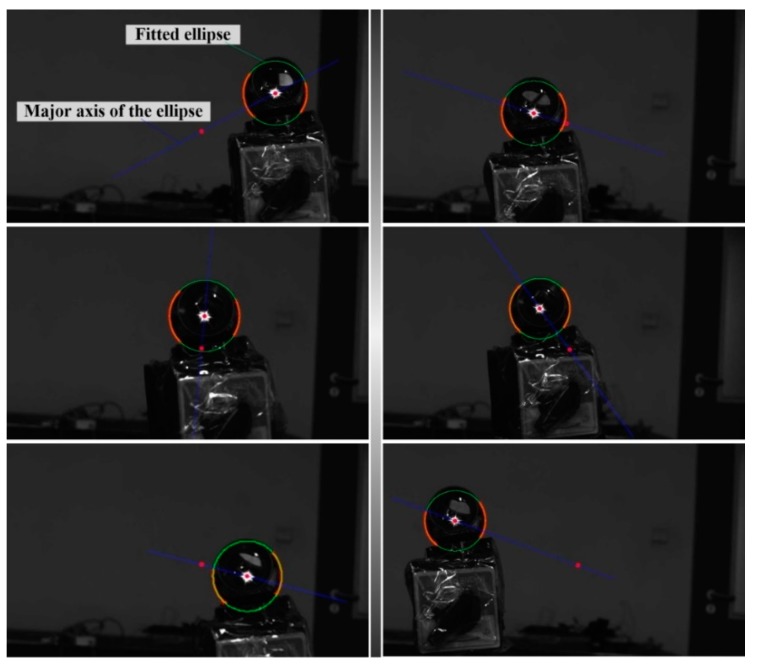
Six examples of ellipse detection and fitting results for the left projection images.

**Figure 4:**
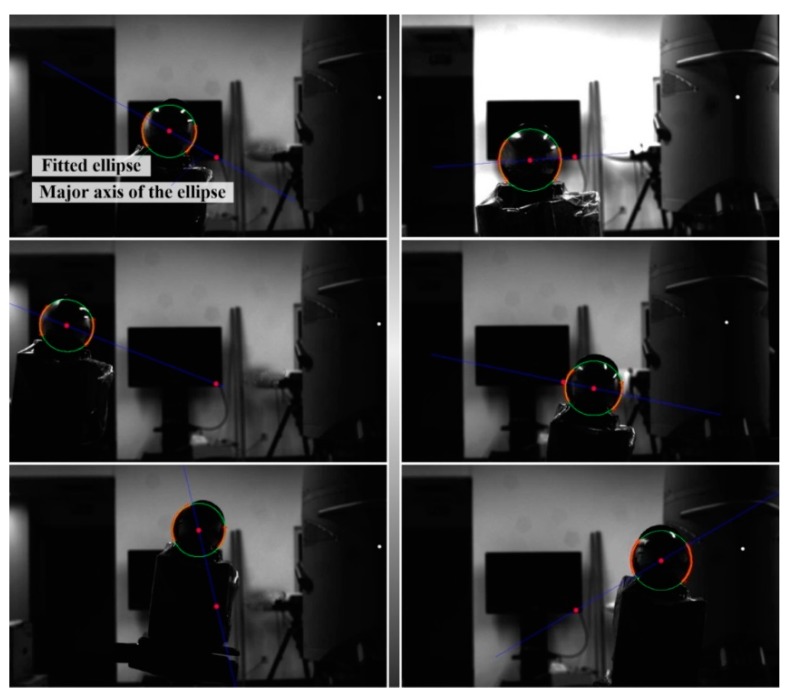
Six examples of ellipse detection and fitting results for the right projection images.

After projection images of the target sphere were captured, edge feature points were selectively extracted using the Harris detector. Then, the ellipse center of the projected sphere was located and the initial estimate of the ellipse equation coefficient was calculated utilizing the method proposed by Bennett et al. [24]. Finally, the parameters of the projection ellipse equation were further optimized using the least square fitting algorithm. Figure 3 and Figure 4 exemplify the ellipse detection and fitting results of left and right projection images, respectively.

It is noteworthy that the distortion of all extracted feature points should be corrected before they are used to locate the ellipse center and further applied to fit the ellipse equation.

### 4.2. 3D Localization of the Target Sphere Center in the LCS and GCS

The intrinsic parameters of the left and right cameras were obtained using the method presented by Zhang [2] and are shown in Table 1.

For each camera, the target sphere was placed at ten different positions, respectively. The 3D coordinates of the target sphere center in the GCS read by the laser tracker and their counterparts in the LLCS and the RLCS that were reconstructed by Equation (4), where R_0_ = 19.5 mm, are shown in Table 2.

### 4.3. Results Analysis and Accuracy Comparison

Given the three-dimensional coordinates of the target sphere center in the GCS and LCS, the initial global calibration of the relative pose between the LLCS and the RLCS can be realized using method described in Section 2.3. After the bundle adjustment based on distance and reprojection constraints, the rectified 3D coordinates of the target sphere center in LLCS and RLCS are shown in Table 3. The extrinsic parameters between LLCS and RLCS before and after the bundle adjustment are shown in Table 4.

**Figure 5:**
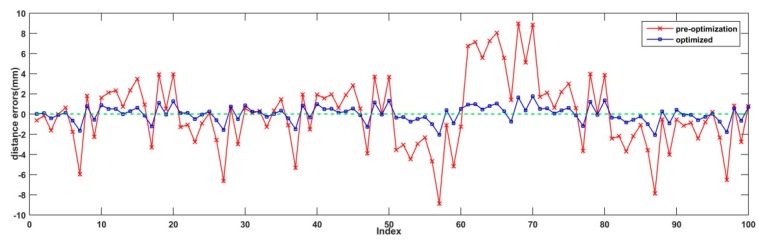
Distance error curves before and after bundle adjustment.

Using distances between the left and right points in the GCS as the reference distances, we can easily compute the corresponding distance errors using points in the LCS. According to the calibration results, the root mean square error of the 100 distances before optimization was about 3.5 mm and that after the bundle adjustment was 0.8 mm. It is notable that the accuracy was increased by two times. The error curves calculated by the points before and after the bundle adjustment are displayed in Figure 5.

**Figure 6:**
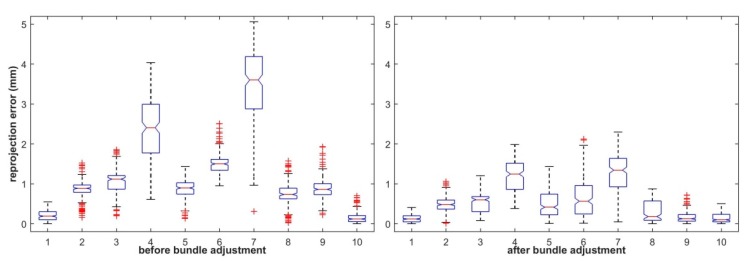
Reprojection errors of the initial and rectified points in the LLCS.

**Figure 7:**
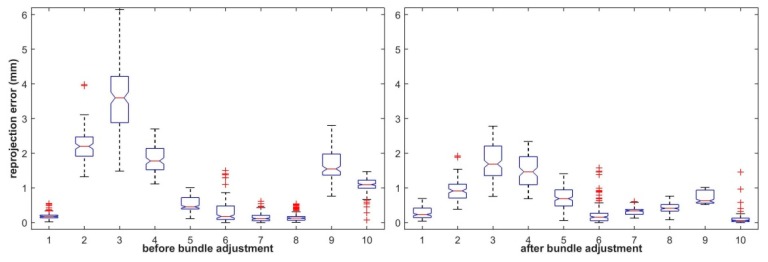
Reprojection errors of the initial and rectified points in the RLCS.

Meanwhile, the reprojection errors using the initial and rectified 3D points in LLCS and RLCS were also calculated and are shown in Figure 6 and Figure 7, respectively. From Figure 6 and Figure 7, we can see that the distance errors between extracted feature points and the reprojection ellipse of each sphere center point after bundle adjustment were all restrained to some extent.

## 5. Conclusions

In this paper, we proposed a flexible and accurate method for global calibration of vision sensors without overlapping fields of view. The presented method utilized the laser tracker to establish the reference coordinate system and an accessory target sphere was used as the calibration medium. By fitting the projection ellipse of the sphere, the three-dimensional coordinates of the target sphere center could be recovered and the initial global calibration can be accomplished. The calibration of extrinsic parameters was further optimized via the bundle adjustment algorithm after the distance and reprojection constraints were introduced. For two point sets with a distance of about 1500 mm apart from each other, the experimental results showed that the distance error could be decreased from 3.5 mm to 0.8 mm after the optimization, which means that the presented method is feasible and flexible for the global calibration of vision sensors, especially when vision sensors are in a confined workspace where common planar targets can be awkward, but a little target sphere is handy.

## Figures and Tables

**Table 1:** Intrinsic parameters of the left and right cameras.

Parameters	*f_x_*	*f_y_*	*u*_0_	*v*_0_	*k*_1_	*k*_2_
Left Camera	2734.41	2734.41	721.84	527.19	−0.228	0.121
Right Camera	1979.14	1979.14	688.91	504.17	−0.129	0.181

**Table 2:** 3D coordinates of the target sphere center in the GCS and LCS (mm).

No.	3D Points within the FOV of Left Camera		3D Points within the FOV of Right Camera	
	Left Points in the GCS	Initial Points in the LLCS	Right Points in the GCS	Initial Points in the RLCS
1	(155.65,2373.49,−403.02)	(−30.19,−34.81,495.24)	(301.44,931.73,−456.51)	(−28.27,2.03,413.56)
2	(158.23,2423.15,−423.13)	(−26.42,−11.00,541.30)	(300.83,832.14,−390.92)	(−25.39,−64.70,515.36)
3	(227.35,2425.11,−407.22)	(44.92,−23.56,542.75)	(382.79,870.49,−411.56)	(−109.86,−42.89,478.41)
4	(105.63,2441.87,-407.39)	(−78.35,−28.65,565.16)	(296.44,825.45,−393.36)	(−20.53,−62.03,519.78)
5	(143.11,2367.60,−431.68)	(−44.53,−6.45,484.90)	(219.94,906.33,−423.91)	(55.39,−32.36,436.12)
6	(211.88,2388.08,−439.87)	(25.59,6.61,499.08)	(254.92,887.01,−459.65)	(20.74,4.51,452.50)
7	(114.49,2401.72,−404.65)	(−70.32,−33.20,519.12)	(308.94,873.64,−435.78)	(−34.56,−19.11,479.23)
8	(135.20,2476.36,−417.43)	(−47.47,−14.78,598.66)	(300.48,910.14,−439.16)	(−26.64,−15.83,437.33)
9	(187.40,2375.10,−417.80)	(1.55,−17.87,491.01)	(288.38,859.78,−398.99)	(−13.25,−56.30,482.57)
10	(163.64,2439.99,−406.56)	(−19.45,−26.72,563.27)	(312.35,856.92,−409.34)	(−37.42,−45.66,488.13)

**Table 3:** Rectified 3D coordinates of the target sphere center in the LLCS and RLCS (mm).

No.	Points in the LLCS		Points in the RLCS	
	Rectified Coordinates	Deviation Vector Δ*P*	Rectified Coordinates	Deviation Vector Δ*P*
1	(−30.18,−34.79,494.07)	(−0.01,−0.02,1.16)	(−27.72,2.06,414.26)	(−0.55,−0.03,−0.70)
2	(−26.51,−11.08,541.53)	(0.09,0.08,−0.23)	(−25.02,−63.81,513.98)	(−0.37,−0.89,1.38)
3	(44.88,−23.40,541.68)	(0.04,−0.16,1.07)	(−108.11,−42.35,478.55)	(−1.75,−0.54,−0.14)
4	(−78.45,−28.44,563.68)	(−0.08,−0.21,1.49)	(−20.48,−61.39,520.22)	(−0.05,−0.64,−0.44)
5	(−44.77,−6.74,485.90)	(0.23,0.29,−1.00)	(54.64,−31.95,436.74)	(0.75,−0.41,−0.62)
6	(25.69,6.33,501.93)	(−0.10,0.29,−2.85)	(20.58,4.24,456.72)	(0.16,0.27,−4.22)
7	(−70.82,−33.43,522.01)	(0.49,0.22,−2.89)	(−33.74,−18.98,473.44)	(−0.82,−0.13,5.79)
8	(−47.40,−14.61,596.78)	(−0.07,−0.17,1.88)	(−26.23,−15.54,436.24)	(−0.42,−0.29,1.09)
9	(1.52,−18.05,492.31)	(0.03,0.18,−1.30)	(−13.32,−56.17,485.05)	(0.07,−0.13,−2.48)
10	(−19.43,−26.55,560.54)	(−0.03,−0.17,2.73)	(−37.13,−45.24,489.14)	(−0.29,−0.43,−1.00)

**Table 4:** Extrinsic parameters between two cameras before and after optimization.

Parameters	*α*	*β*	*γ*	*t_x_*	*t_y_*	*t_z_*
Pre-optimization	−0.0496	3.0921	−0.3114	36.6868	−192.5890	−525.9032
Optimized	−0.0392	3.1358	−0.1498	31.2351	−83.7498	−538.1350
